# Impact of FasL Stimulation on Sclerostin Expression and Osteogenic Profile in IDG-SW3 Osteocytes

**DOI:** 10.3390/biology10080757

**Published:** 2021-08-07

**Authors:** Adela Kratochvilova, Alice Ramesova, Barbora Vesela, Eva Svandova, Herve Lesot, Reinhard Gruber, Eva Matalova

**Affiliations:** 1 Laboratory of Odontogenesis and Osteogenesis, Institute of Animal Physiology and Genetics, Academy of Sciences, 60200 Brno, Czech Republic; 21506@mail.muni.cz (A.K.); ramesovaa@vfu.cz (A.R.); veselab.lab@gmail.com (B.V.); 184576@mail.muni.cz (E.S.); herve.lesot@gmail.com (H.L.); 2 Department of Oral Biology, University Clinic of Dentistry, Medical University Vienna, Sensengasse 2a, 1090 Vienna, Austria; reinhard.gruber@meduniwien.ac.at; 3 Institute of Physiology, Faculty of Veterinary Medicine, Veterinary University Brno, 61200 Brno, Czech Republic

**Keywords:** bone, Fas/FasL signalling, caspases, osteocyte differentiation, non-apoptotic

## Abstract

**Simple Summary:**

FasL used to be considered as a classical ligand triggering cell death (apoptosis) via its receptor, Fas and thefollowing caspase cascade. As such, it is known to be involved in regulation within the bone. Recently, however, the knowledge has expanded about the non-apoptotic and caspase-independent engagement of the Fas/FasL pathway. The present investigation identified that stimulation of osteocytic IDG-SW3 cells by FasL leads to a dramatic decrease in expression of the major osteocytic marker, sclerostin. Additionally, other key components of the osteogenic pathways were impacted, notably in a caspase-independent manner. Such findings are of importance for basic biology as well as biomedical applications since osteocytes are the major population within adult bones and Fas signalling is one of therapeutical targets, e.g., in the anti-osteoporotic treatment.

**Abstract:**

The Fas ligand (FasL) is known from programmed cell death, the immune system, and recently also from bone homeostasis. As such, Fas signalling is a potential target of anti-osteoporotic treatment based on the induction of osteoclastic cell death. Less attention has been paid to osteocytes, although they represent the majority of cells within the mature bone and are the key regulators. To determine the impact of FasL stimulation on osteocytes, differentiated IDG-SW3 cells were challenged by FasL, and their osteogenic expression profiles were evaluated by a pre-designed PCR array. Notably, the most downregulated gene was the one for sclerostin, which is the major marker of osteocytes and a negative regulator of bone formation. FasL stimulation also led to significant changes (over 10-fold) in the expression of other osteogenic markers: Gdf10, Gli1, Ihh, Mmp10, and Phex. To determine whether these alterations involved caspase-dependent or caspase-independent mechanisms, the IDG-SW3 cells were stimulated by FasL with and without a caspase inhibitor: Q-VD-OPh. The alterations were also detected in the samples treated by FasL along with Q-VD-OPh, pointing to the caspase-independent impact of FasL stimulation. These results contribute to an understanding of the recently emerging pleiotropic effects of Fas/FasL signalling and specify its functions in bone cells.

## 1. Introduction

Fas (CD95) and FasL (CD95L, CD178) are known particularly from the immune system [1,2] and activation of the extrinsic apoptotic pathway [3]. FasL is a ligand for the Fas receptor. It belongs to the TNF family and is mainly synthesised as a membrane-bound protein. In apoptosis, the Fas receptor oligomerizes upon binding of FasL and recruits the Fas-associated protein with a corresponding death domain (FADD). FADD interacts with the apical caspase-8 to switch on the intracellular caspase cascade, representing canonical Fas/FasL signalling [4].

Additionally, there is increasing evidence that Fas/FasL can trigger caspase-independent cell death or even interfere with processes beyond cell death [5,6]. One mechanism to explain the diverse effects of FasL is the cleavage of the transmembrane ligand by metalloproteinases, making it soluble. The soluble FasL (sFasL) is also expected to drive the cells into non-apoptotic fates; however, the relevant pathways involved in this have not yet been elucidated [7].

Fas/FasL signalling in the bone has been investigated for decades with a respect to physiological maintenance as well as pathological disorders [8,9]. In the latter case, osteoporosis has captured the most interest, since osteoblast-induced osteoclast apoptosis is one of the possible targets in anti-osteoporotic therapies [10,11,12]. Therefore, the impact of Fas/FasL in osteoblasts and osteoclasts, their interactions, and possible modulations of Fas/FasL signalling have been in focus [13]. Much less attention has been paid to osteocytes, cells which are prevalent in the mature bone.

Despite their embedding in the calcified extracellular matrix, osteocytes actively participate in bone-related molecular networks [14]. Osteocytes are even considered as the master cells orchestrating the communication between osteoblasts and osteoclasts, and regulating the bone multicellular unit [15]. Whether adult bone homeostasis is controlled by Fas-mediated apoptosis is still unclear [16]. Nevertheless, FasL conditional knockout (osteoblast-specific) displays an osteopenic phenotype [9], with a significantly decreased number of apoptotic osteoclasts. Additionally, it has been assumed that Fas/FasL interactions may contribute to the maintenance of a proper number of mature osteocytes since osteocytes express Fas receptors [17]. Unfortunately, there is a lack of evidence about the effects of FasL on osteocytes. This might be due to difficult access to these cells in vivo compared with osteoblasts and osteoclasts, along with the very limited repertoire of osteocytic cell lines and the demands of handling these in vitro [18]. 

One of the available osteocytic cell lines is IDG-SW3, considered to be the best model to work with mature Sost-positive osteocytes [18]. In this research, advantage was taken of this system to test the impact of FasL stimulation on the osteogenic profile of the IDG-SW3 osteocytes. Moreover, to follow the caspase-independent pathways, a general caspase inhibitor (Q-VD-OPh) was applied in parallel cultures.

## 2. Materials and Methods

### 2.1. Cell Line and Culture

The cell line IDG-SW3 (EKC001) derived from mouse long bones was obtained from Kerafast (Boston, MA, USA) and expanded in favourable conditions (33 °C, 5% CO_2_) in a proliferation medium consisting of MEM alpha (Gibco, Waltham, MA, USA), 10% fetal bovine serum (Sigma-Aldrich, Burlington, MA, USA), 1% penicillin/streptomycin (Sigma-Aldrich), and 5 ng/mL mouse IFN-gamma recombinant protein (Gibco). To induce osteocyte differentiation, cells were plated at a density of 4 × 10^4^ cells/cm^2^; once the cells reached confluence, the culture conditions were changed (37 °C, 8% CO_2_) and an IFN-gamma free medium enriched with 50 μg/mL ascorbic acid (AA) and 4 mM β-glycerol phosphate (βGP) was used. Cells were cultured on rat tail Type I collagen (Gibco)-coated plates and the medium was replaced every 3 days.

IDG-SW3 osteocytes were used in the experiment as the most suitable line expressing high levels of sclerostin upon differentiation [19]. Three days before completed differentiation (Day 28), the medium was supplemented with 150 ng/mL of recombinant human sFas ligand (310-03H, Peprotech, Cranbury, NJ, USA) or with a combination of sFas ligand and the general caspase inhibitor Q-VD-OPh (OPH001, R&D Systems, Minneapolis, MN, USA) at a concentration of 100 μM (FasL + OPh). The doses used in the investigation were selected based on published data [13,20]. The control group without any supplementation and the control group with OPh only (100 μM) were run in parallel in 3 independent experimental sets. Cells were harvested after 72 h of treatment; the medium was changed once during this time. Overall, cells were differentiated for 28 days, as commonly used within protocols [19]. 

Calvarial cells were obtained and differentiated as described in [21] for 16 days. The concentrations applied and the experiment time course were the same as in the case of the IDG-SW3 cells.

### 2.2. Cell Staining and Immunocytofluorescence

To test cell viability, differentiated and treated IDG-SW3 cells were stained by 0.4% Trypan Blue (Gibco).

For immunocytofluorescence, IDG-SW3 cells were cultured on histological slides, fixed in 4% PFA, washed in PBS, and treated with 0.1% Triton X-100 for 15 min. The cleaved caspase-3 primary antibody (9664, Cell Signaling, Danvers, MA, USA) was diluted 1:50 and applied overnight at 4 °C. Alexa Fluor 488 (A11034, Thermo Fischer Scientific, Waltham, MA, USA) was diluted 1:200 and then applied for 40 min at RT. Nuclei were visualised by ProLong Gold Antifade reagent with DAPI (Thermo Fischer Scientific).

Positive control of apoptosis (Figure S1) was achieved by stimulation of the cells by doxorubicin (5927, Cell Signaling) at a concentration of 5 µM for 6 h as recommended by the manufacturer. 

### 2.3. TUNEL

Detection of apoptotic cells was performed by a TUNEL assay (S7100, Merck Millipore, Burlington, MA, USA) according to the instructions of the manufacturer.

### 2.4. RNA Isolation, Real-Time PCR, PCR Arrays

Cells for RNA isolation were lysed in 900 μL of QIAzol Lysis Reagent and RNA was isolated by the RNeasy Plus Universal Kit (Qiagen, Hilden, DE). cDNA was prepared using Super Script VILO (Thermo Fisher Scientific). qPCR was performed in 10 μL of a final reaction mixture containing the 1-step GB Ideal PCR Master Mix (Generi Biotech, Hradec Kralove, Czech Republic). The TaqMan Gene Expression Assay (Thermo Fisher Scientific) was applied for detecting the gene expression of *Dmp1* (Mm01208363_m1), *Gdf10* (Mm01220860_m1), *Gli1* (Mm00494654_m1), *Ihh* (Mm00439613_m1), *Mmp10* (Mm01168399_m1), *Phex* (Mm00448119_m1), and *Sost* (Mm00470479_m1). The expression levels were calculated using the ΔΔCT method with normalisation based on actin levels (*Actb*, Mm02619580_g1). Osteogenic-related gene expression was detected by RT2 Profiler PCR Array Mouse Osteogenesis (PAMM026Z, Qiagen). The format included positive and negative controls and the set of housekeeping genes (*Actb*, *B2m*, *Gapdh*, *Gusb*, and *Hsp90ab1*).

### 2.5. Statistical Analysis

PCR array data were statistically evaluated by Qiagen Gene Globe, as recommended by the manufacturer (Qiagen Gene Globe. Available online: https://geneglobe.qiagen.com/us/, accessed on 6 August 2021). Statistical significance was determined as *p* < 0.05; the threshold of fold regulation was ±2. Three biological replicates were evaluated in each group. Real-time PCR expression levels were calculated using the ∆∆CT method and the results were analysed using a 2-tailed *t*-test. Reactions were performed in triplicate for each sample. 

## 3. Results

### 3.1. Maturation of IDG-SW3 Osteocytes

**Figure 1:**
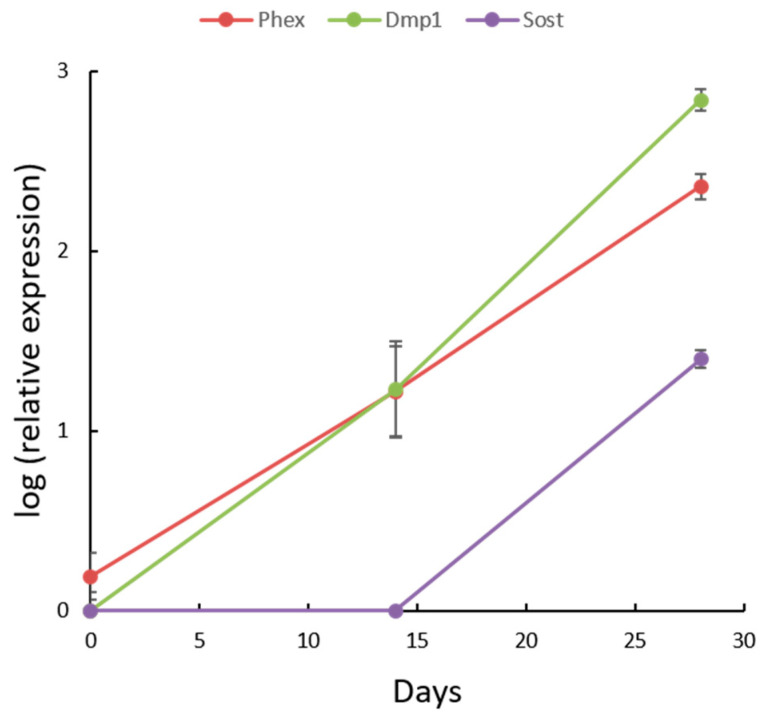
Time-dependent expression of osteogenic markers *Phex*, *Dmp1*, and *Sost* at three intervals (Days 0, 14, 28) within the course of IDG-SW3 differentiation.

To confirm the maturation of IDG-SW3 osteocytes, the expression of key markers of differentiation (*Phex*, *Dmp1*, and *Sost*) was evaluated by qPCR after 0, 14, and 28 days of culturing in the differentiation medium (Figure 1). The expression of *Phex* and *Dmp1* increased with the course of differentiation. *Sost* expression appeared from the second half of the differentiation process and further on, with an increasing tendency, which corresponded to the curve expected from the differentiation protocol. 

### 3.2. FasL Impacts the Osteogenic Profile of IDG-SW3 Cells

**Figure 2:**
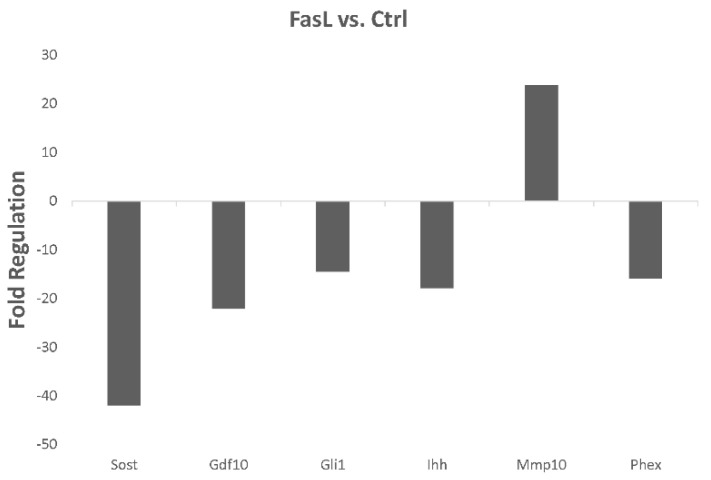
PCR array analysis of osteogenesis-related gene expression in the differentiated IDG-SW3 cells treated with FasL compared with untreated controls. Only statistically significant changes (*p* ≤ 0.05) with more than +/– 10-fold regulation are shown.

To evaluate the impact of FasL on mature sclerostin-expressing IDG-SW3 osteocytes, soluble FasL was applied in the medium for 72 h. Comparison of the osteogenic expression profile with the controls indicated significant alterations in several important markers (Figure 2). Notably, there was a striking decrease in sclerostin (*Sost)*, the major marker of osteocytes. In this case, the fold regulation dropped by more than 40 times in the FasL-treated cells (*Sost*, fold regulation: −41.97, *p* < 0.001). Among the genes with more than 10-fold altered expression, we also detected *Gdf10* (growth differentiation factor 10; fold regulation: −22.03, *p* = 0.019), *Gli1* (GLI-Kruppel family member GLI1; fold regulation: −14.43, *p* < 0.001), *Ihh* (Indian hedgehog; fold regulation: −17.81, *p* < 0.001), *Mmp10* (Matrix metalloproteinase 10; fold regulation: 23.84, *p* < 0.001), and *Phex* (Phosphate-regulating gene with homologies to endopeptidases on the X chromosome; fold regulation: −15.90, *p* < 0.001). Alterations in several other factors reached at least double the threshold (Table S1).

Additionally, a pilot experiment was run in primary calvarial cells (Figures S1 and S2). The Sost expression level in these cells after differentiation was significantly lower than in the case of IDG-SW3 (Figure S1A). Notably, Fas receptor expression in the calvarial cells was dramatically lower than in the IDG-SW3 cells (Figure S1B). This may be the reason why the decrease in Sost expression after FasL stimulation was not as massive as in the case of IDG-SW3 cells (Figure S2A). Nevertheless, the expression of Sost, as well as of the other genes (Gdf10, Gli, Ihh, and Phex) downregulated in IDG-SW3 cells, also dropped in the calvarial cells (Figure S2A–E).

### 3.3. FasL in Osteocytes Stimulates Caspase-Independent Signalling

**Figure 3:**
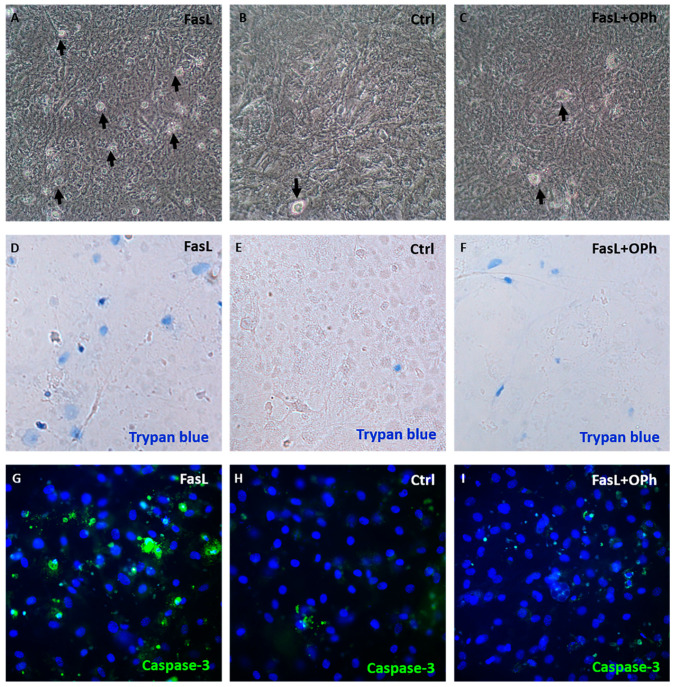
The microscopic appearance of differentiated IDG-SW3 cells after FasL stimulation (**A**), untreated controls (**B**), and cells after treatment with FasL and OPh (**C**). Black arrows indicate cells with an apoptotic morphology. Trypan blue staining: IDG-SW3 cells after FasL stimulation (**D**), untreated controls (**E**), and cells after treatment with FasL and OPh (**F**). Blue cells are non-viable. Caspase-3 activation: IDG-SW3 cells after FasL stimulation (**G**), untreated controls (**H**), and cells after treatment with FasL and OPh (**I**). Positive signals are in green; nuclei were counterstained by DAPI (blue).

To distinguish between the canonical pathway (caspase cascade) and the potentially caspase-independent impact of FasL, the Q-VD-OPh (OPh) general caspase inhibitor was used. Controls, FasL-stimulated, and FasL + OPh-treated cells were run in parallel. All experimental groups displayed a deposition of extracellular matrix during the differentiation process. In the case of FasL treatment, an increased number of apoptotic/non-viable cells was evident (Figure 3A,D,G) compared with the control group (Figure 3B,E,H). Quantification of positive cells confirmed the increased number of non-viable cells (Figure S3A). Positive control of cell viability (Figure S3B–E) and apoptosis (Figure S3F,G) was performed in doxorubicin-treated samples. In the case of cells treated with FasL plus OPh, the analysis similarly revealed the reduced impact of FasL on apoptosis induction/viability (Figure 3C,F,I).

**Figure 4:**
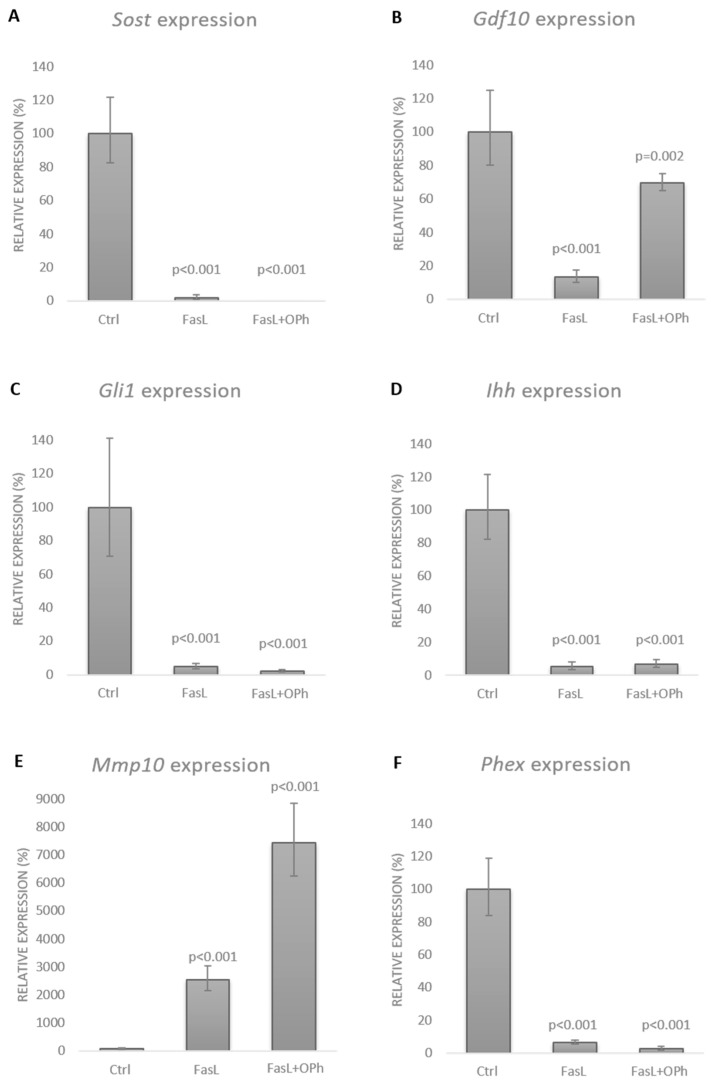
Expression of *Sost* (**A**), *Gdf10* (**B**), *Gli1* (**C**), *Ihh* (**D**), *Mmp10* (**E**), and *Phex* (**F**) in the differentiated IDG-SW3 cells after FasL stimulation with (FasL + OPh) and without (FasL) caspase inhibition. Expression levels were compared with those in the untreated controls. The results are shown in %, indicating the mean ± standard deviation of three replicates (expression in the control cells was set to 100%).

As expected, the OPh inhibitor effectively blocked the apoptotic caspase activation effect of FasL. Comparison of the FasL and FasL + OPh groups by qPCR analysis revealed the same effect on the expression of sclerostin as well as the other genes, with expression altered by at least 10-fold after FasL stimulation (Figure 4). Based on the qPCR data, the expression of Sost dropped to 2% in the FasL group compared with the control and to an undetectable level in FasL + OPh compared with the control (Figure 4A). The expression of Gdf10 decreased to 13% in FasL and to 70% in the FasL + OPh group (Figure 4B). Expression of Gli1 decreased to 5% in the case of FasL treatment and to 2% after FasL + OPh (Figure 4C). Similarly, the expression of Ihh decreased to 6% and to 7% after FasL and FasL + OPh treatment, respectively (Figure 4D). An increase in expression was detected for Mmp10, which rose to 2566% after FasL and even to 7439% after FasL + OPh treatment (Figure 4E). However, this dramatic increase was affected by the very low expression of Mmp10 in untreated cells. The expression of Phex decreased to 7% after FasL treatment and to 3% after FasL + OPh (Figure 4F). 

**Figure 5:**
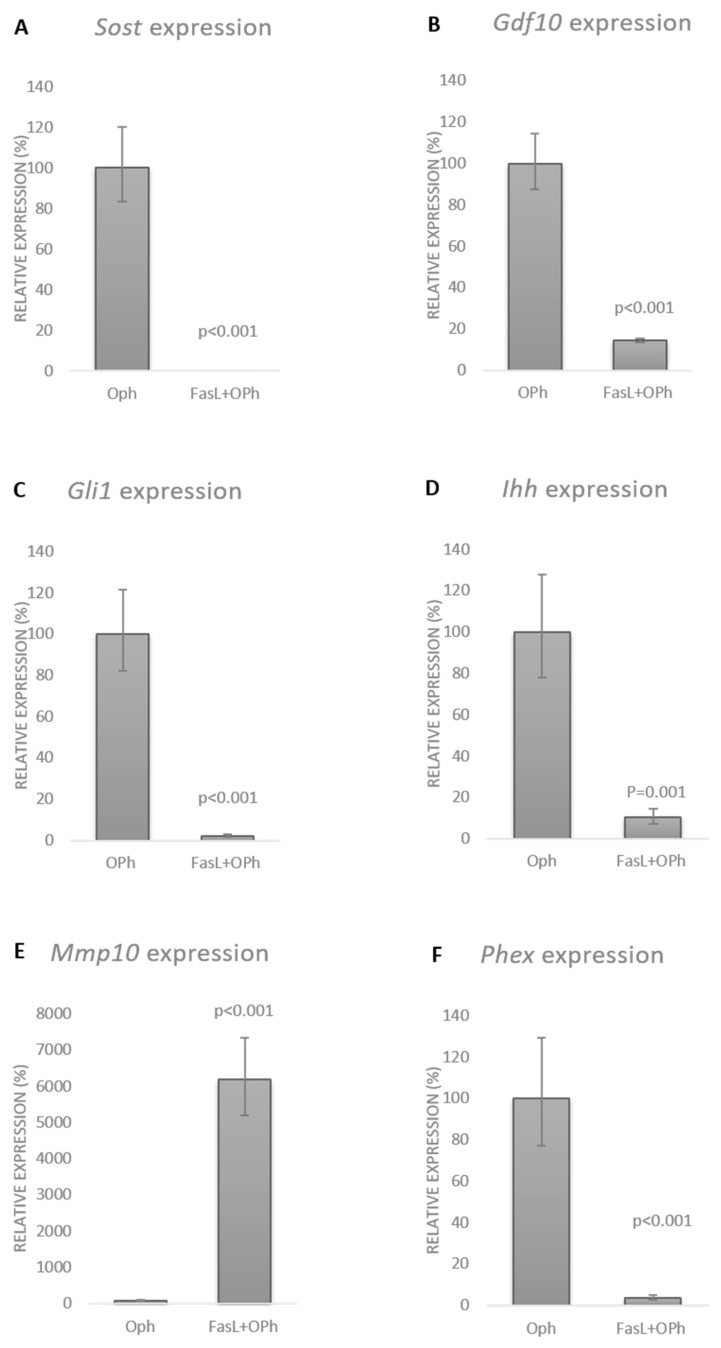
Expression levels of cells treated with (FasL + OPh) compared with those treated with OPh only (*Sost*, *Gdf10*, *Gli1, Ihh*, *Mmp10*, and *Phex*, respectively) (**A**–**F**). The results are shown in %, indicating the mean ± standard deviation of three replicates (expression in the control cells was set to 100%).

An additional control, treated by OPh only, was performed and is displayed for the expression of each gene in comparison with the FasL + OPh samples: *Sost* (Figure 5A), *Gdf10* (Figure 5B), *Gli1* (Figure 5C), *Ihh* (Figure 5D), *Mmp10* (Figure 5E), and *Phex* (Figure 5F).

## 4. Discussion

The major finding indicated by our results is that FasL stimulation of IDG-SW3 osteocytes negatively impacts sclerostin expression and that the mechanism is caspase-independent. Sclerostin is the key marker of osteocytes [22], acting as an inhibitor of bone formation via interference with the Wnt signalling pathways [23]. This results in the inhibition of osteoblastic activity and bone formation, along with activation of the osteoclasts and increased bone resorption [22]. There have been several receptor-mediated pathways reported in Sost regulation, such as stimulation by PTH, TGFβ, TNFα, or BMP with the subsequent intracellular pathways [24]. The mechanism mediating the effect of FasL/Fas on Sost expression is not known.

Despite the experiments being performed in a specific cell line (highly Sost-expressing), and thus generalisation will need further confirmation, the data provide solid initial evidence about the participation of FasL/Fas pathways in Sost regulation, at least in IDG-SW3 osteocytic cells. The new observation related to the effect of FasL stimulation in osteocytes matches with the findings in FasL conditional knockout (osteoblast-specific) displaying an osteopenic phenotype [9]. Sost expression in these mice has not yet been evaluated. The only indication pointing to the impact on Sost expression in *gld* mice was associated with the very early prenatal bone development when the first transition of osteoblasts into osteocytes occurs [25]. Notably, the pre- and postnatal bone phenotypes of the *gld* mice differed, and it was hypothesised that the major reason could be a change in the proportion of osteoblasts and osteocytes towards mature (adult) bone [26]. Our results from the FasL stimulation experiments in IDG-SW3 cells support the hypothesis that in vivo, the impact of FasL deficiency depends on the osteoblast/osteocyte ratio and thus the sclerostin expression levels. Additionally, the findings are in agreement with the most recent results from adult *gld* mice, where FasL deficiency was associated with the impaired healing of extraction sockets [27].

Osteoblasts are the major source of FasL within the bone [25]. Their membrane-bound FasL can interact with osteoclasts by direct contact [9], which is limited within the osteocyte network embedded by the calcified extracellular matrix. The soluble FasL, working in a paracrine manner, has a broader spectrum of possible interactions, including osteocytes. Based on recent findings, the soluble form of FasL is the form working in non-apoptotic signalling or at least in caspase-independent pathways [5]. This conclusion is in agreement with our observations when the inhibition of caspases did not interfere with the modulations in the gene expression of the affected osteogenic markers after FasL stimulation. 

FasL solubilisation happens in vivo as a result of oestrogen-induced MMP3 cleavage of FasL in osteoblasts [8]. The interaction of FasL with the Fas receptor on osteoclasts caused a reduced osteoclast number when treated by oestrogen [28]. FasL is thus considered a target in post-menopausal osteoporosis therapies [9,11]. Neutralising antibodies blocking FasL signalling could not only positively impact the osteoblast/osteoclast network but could apparently also interfere with sclerostin levels in osteocytes.

Along with sclerostin expression, the FasL stimulation caused significant (over 10-fold) changes in the transcription of the genes for Gdf10, Gli1, Ihh, Mmp10, and Phex.

Gdf10 (also named BMP3) decreased after FasL stimulation and has multiple roles in skeletal morphogenesis [29]. Gdf10 inhibits the differentiation of osteoblasts via the SMAD2/3 pathway [30]. As such, stimulation by FasL that caused decreased Gdf10 expression would contribute to osteoblastic differentiation. 

Formation of the extracellular matrix accompanying the transition of osteoblasts into osteocytes is also mediated by hedgehog signalling and its target, Gli1 [31]. Ihh and Gli1 were among the genes showing decreased expression after FasL stimulation. Ihh is important in bone development, particularly in the endochondral type, together with PTHrP and Runx2 [32]. Recently, PTH1R activation was proposed to induce pro-survival actions via primary cilia- and Gli-1-dependent mechanisms and to modulate osteogenic responses via primary cilia-dependent and Gli-1-independent pathways in osteocytes and osteoblasts [33]. Whether FasL could be involved in this fine-tuning is questionable. Nevertheless, among the different signalling mechanisms stimulated by primary cilia, the hedgehog pathway has been reported in the regulation of bone development [34].

Moreover, Mmp10, which increased after FasL stimulation, could contribute to this process, since the spectrum of matrix metalloproteinases roles includes the differentiation of osteoblasts, bone formation, and the transition of osteoblasts into osteocytes [35,36]. The position of Mmp10 to apoptosis is unclear, but some protective functions from the protein kinase C/p53-induced apoptosis have been described [37]. Overexpression of Mmp10 was associated with a reduction in FasL and cleaved caspase-8 [38]. Notably, Mmp10, despite being undetectable or at a very low level in most healthy tissues, is readily induced in response to injury or inflammatory stimuli and participates in the calcification process and bone repair [39].

FasL stimulation impacts important molecules related to the osteoblast–osteocyte transition and thus the expression of major osteocytic markers, particularly Phex and sclerostin.

The monoclonal antibody against sclerostin (romosozumab) is in Phase 3 development for potential anti-osteoporotic strategies [40,41]. This treatment was reported to be more effective when combined with anti-resorptive therapies, where interference with Fas/FasL has also been considered [42]. The FasL-mediated decrease in Sost, as indicated in the present research, along with the well-known FasL-mediated apoptosis of osteoclasts sounds like a challenging double benefit. 

Nevertheless, in this context, the systemic impact must be considered since osteocytes not only impact other bone cell types but also distant organs [40]. The major secreted hormone-like molecules include sclerostin and FGF23. FGF23 impairs Vitamin D metabolism and impacts phosphate and calcium balance [43]. This effect is reinforced by the cleavage of osteopontin, which inhibits bone mineralisation through FGF23 [44]. Notably, the production of FGF23 is regulated by Phex, which is also produced by osteocytes [45] and was impacted in our investigation. 

Therefore, interference with the expression of osteocytic markers working in a paracrine as well as an endocrine manner must be considered. This also applies to alternative non-canonical pathways, such as caspase-independent FasL mediated signalling. 

The non-apoptotic pathways that utilise Fas activating signals are, in general, poorly understood, despite evidence about the cell-death independent activities of the Fas/FasL complex accumulating [5,46,47,48,49]. The possible downstream mechanism triggering FasL signalling towards the apoptotic vs. the non-apoptotic fate is not known. The Fas receptor has no enzymatic activity; therefore, mechanisms such as fine-tuned control of its aggregation/conformation, post-translational modifications, or changes in the distribution pattern within the membrane are being considered [48,49]. Interference with NF-kB signalling is another option; however, the data are still controversial. In particular, the caspase-dependent vs. caspase-independent manner is not yet clear [47]. The induction of mitogen-activated protein kinase (MAPK) cascades has been investigated, including caspase-independent MAPK stimulation. Kinases as promising candidates for Fas/FasL-initiated decision-making between cell survival and death have also been identified in the latest research [50]. 

Other types of cell death must be considered within the final molecular concept, particularly autophagy. The participation of autophagy in FasL/Fas signalling has been reported in several systems [51,52,53]. Notably, caspases have even been considered as an important switch between autophagy and apoptosis [54]. While the present study aimed to investigate caspase-dependent and -independent FasL/Fas signalling in general, further investigations will have to be designed to distinguish the pathways associated with apoptosis vs. autophagy. Apoptosis and autophagy are involved in bone physiology as well as pathophysiology [55,56], but the question of the possible link via specific caspases remains open for further research.

The present study was performed in IDG-SW3 osteocytic cells, a specific line with a high expression of sclerostin. Despite some limitations, this model allowed for novel achievements in the specification of the non-canonical FasL signalling related to osteogenesis. Recently, more evidence has accumulated for the role of osteocytes in bone signalling [36], and their transcriptome signature is being deciphered [57] to understand the role of these cells in skeletal homeostasis and disease. Simultaneously, 3D culture systems (including IDG-SW3 cells) are being developed to examine particular points in vitro, which would overcome certain difficulties with the isolation, culturing, and differentiation of osteocytes [18]. Such an in vitro system would also allow for better extrapolation to the in vivo situation [58]. In this context, further investigation of the pleiotropic effects of Fas/FasL signalling in bone cells is a challenging issue.

## 5. Conclusions

This investigation provided the first evidence about the impact of FasL/Fas signalling on the expression of sclerostin and other osteogenic factors in osteocytic cells and indicated a caspase-independent mechanism.

## Data Availability

There are no additional data presented outside of the manuscript.

## Supplementary Materials

The following are available online at https://www.mdpi.com/article/10.3390/biology10080757/s1, Table S1: PCR Array analysis of osteogenesis-related gene expression in the differentiated IDG-SW3 cells treated with FasL compared to untreated controls. Blue marked genes: significantly changed, more than 2-fold, *p* ≤ 0.05. Red marked genes: significantly changed, more than 10-fold, *p* ≤ 0.05, Figure S1: Comparison of Sost (A) and Fas (B) expression in IDG-SW3 cells and calvarial primary cells, Figure S2: Expression of Sost (A), Gdf10 (B), Gli1 (C), Ihh (D), and Phex (E) in calvarial cells after FasL stimulation, Figure S3: Quantification of non-viable IDG-SW3 cells in control, FasL and FasL+OPh treated cultures counted as a percentage of trypan blue-positive cells, cells were counted in four independent fields of vision (A). Positive control of apoptosis was performed by treatment of 5 μM doxorubicin (B, C). After 6 h, treated cells (C) began to die, left the surface, and floated in the medium. The remaining doxorubicin-treated cells showed decreased viability (E) compared to untreated cells (D). To confirm ongoing apoptosis, TUNEL assay, a method for detection of more pronounced stages of apoptosis, was used (F, G). Ctrl: control, Dox: doxorubicin, OPh: Q-VD-OPh inhibitor.
