# Supplementary material for: Impact of FasL Stimulation on Sclerostin Expression and Osteogenic Profile in IDG-SW3 Osteocytes
Source: Biology (Basel). 2021 Aug 7;10(8):757. doi: 10.3390/biology10080757 (PMC8389703; doi:10.3390/biology10080757)
Supplement: Supplementary file 1 [file biology-10-00757-s001.zip › biology-1305343-supplementary.pdf]

Gene	Fold Regulation	p-value	Symbol	Fold Regulation	p-value
Acvr1	1.22	0.001731	**Ihh**	**-17.81**	**0.000047**
Ahsg	-4.79	0.083925	**Itga2**	**-8.14**	**0.000231**
**Alpl**	**-3.85**	**0.000060**	Itga2b	-1.34	0.077427
Anxa5	1.04	0.227046	Itga3	1.37	0.009309
**Bglap**	**-8.46**	**0.000023**	Itgam	1.62	0.313404
Bgn	-1.52	0.000189	Itgav	-1.10	0.024203
Bmp1	1.16	0.245847	Itgb1	1.01	0.828295
**Bmp2**	**3.76**	**0.000516**	**Mmp10**	**23.84**	**0.000001**
Bmp3	-1.59	0.000369	Mmp2	-1.25	0.015882
Bmp4	-1.33	0.009716	Mmp8	-1.05	0.697895
**Bmp5**	**-4.66**	**0.000091**	Mmp9	1.02	0.981477
Bmp6	-1.69	0.017591	Nfkb1	1.53	0.000945
Bmp7	-1.57	0.346457	**Nog**	**-2.70**	**0.003134**
Bmpr1a	-1.28	0.000305	Pdgfa	-1.59	0.002023
**Bmpr1b**	**8.10**	**0.000091**	**Phex**	**-15.90**	**0.000030**
Bmpr2	1.21	0.040442	Runx2	-1.26	0.001041
**Cd36**	**-4.21**	**0.000884**	Serpinh1	-1.74	0.000325
Cdh11	-1.29	0.000465	Smad1	-1.21	0.003300
**Chrd**	**-2.30**	**0.000067**	Smad2	1.07	0.088414
**Col10a1**	**-2.29**	**0.000084**	Smad3	1.24	0.003826
**Col14a1**	**-4.92**	**0.000078**	Smad4	-1.32	0.318705
**Col1a1**	**-2.88**	**0.000341**	Smad5	1.09	0.111228
**Col1a2**	**-2.86**	**0.000002**	**Sost**	**-41.97**	**0.000263**
**Col2a1**	**-2.74**	**0.009954**	Sox9	-1.09	0.296557
Col3a1	-1.31	0.004630	**Sp7**	**-3.03**	**0.000102**
Col4a1	-1.27	0.010595	**Spp1**	**2.89**	**0.009234**
Col5a1	-1.01	0.865812	Tgfb1	-1.06	0.341045
**Comp**	**-6.69**	**0.000011**	Tgfb2	-1.46	0.000391
**Csf1**	**3.34**	**0.000009**	Tgfb3	-1.15	0.171414
Csf2	-6.17	0.098221	Tgfbr1	-1.26	0.057758
Csf3	-1.89	0.205988	**Tgfbr2**	**2.18**	**0.000505**
Ctsk	-1.23	0.007999	Tgfbr3	1.85	0.000931
**Dlx5**	**-3.34**	**0.000037**	Tnf	2.05	0.148709
Egf	-1.60	0.015231	Tnfsf11	1.48	0.060862
Fgf1	-1.32	0.035594	Twist1	1.04	0.383083
Fgf2	-1.40	0.000010	Vcam1	1.90	0.000018
Fgfr1	-1.11	0.062306	Vdr	-1.52	0.001549
**Fgfr2**	**-3.77**	**0.000034**	Vegfa	-1.39	0.004909
Flt1	1.55	0.128908	Vegfb	1.19	0.018593
Fn1	1.70	0.000381	Actb	1.05	0.278607
**Gdf10**	**-22.03**	**0.000026**	B2m	-1.30	0.009439
**Gli1**	**-14.43**	**0.000017**	Gapdh	-1.22	0.018293
Icam1	1.45	0.000608	Gusb	1.33	0.004125
**Igf1**	**-2.25**	**0.000014**	Hsp90ab1	1.14	0.066021
Igf1r	1.28	0.022135			

Table S1. PCR Array analysis of osteogenesis-related gene expression in the differentiated IDG-SW3 cells treated with FasL compared to untreated controls. Blue marked genes: significantly changed, more than 2-fold, $p \leq 0.05$. Red marked genes: significantly changed, more than 10-fold, $p \leq 0.05$.

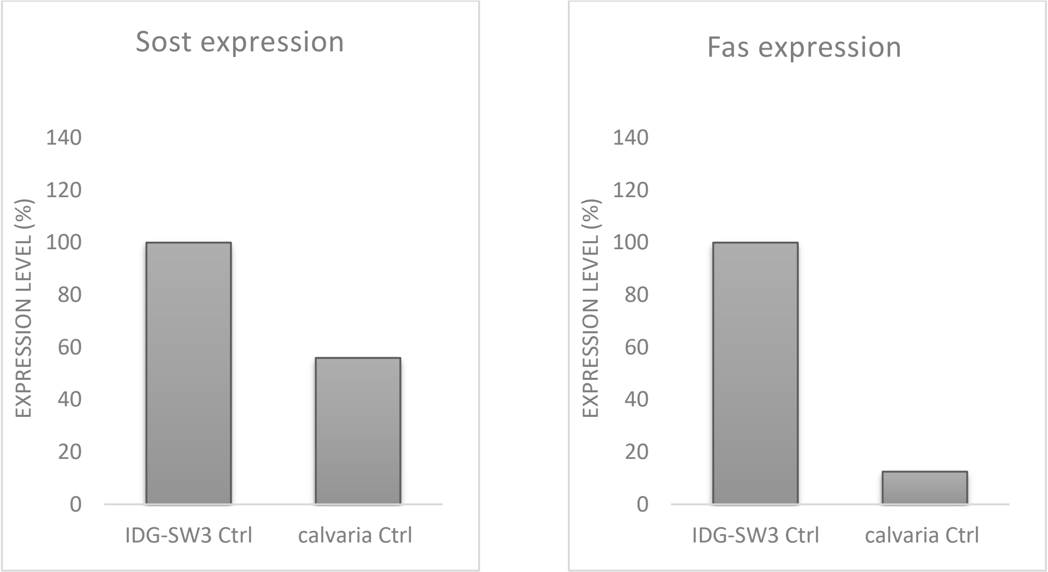

Figure S1: Comparison of Sost (A) and Fas (B) expression in IDG-SW3 cells and calvarial primary cells.

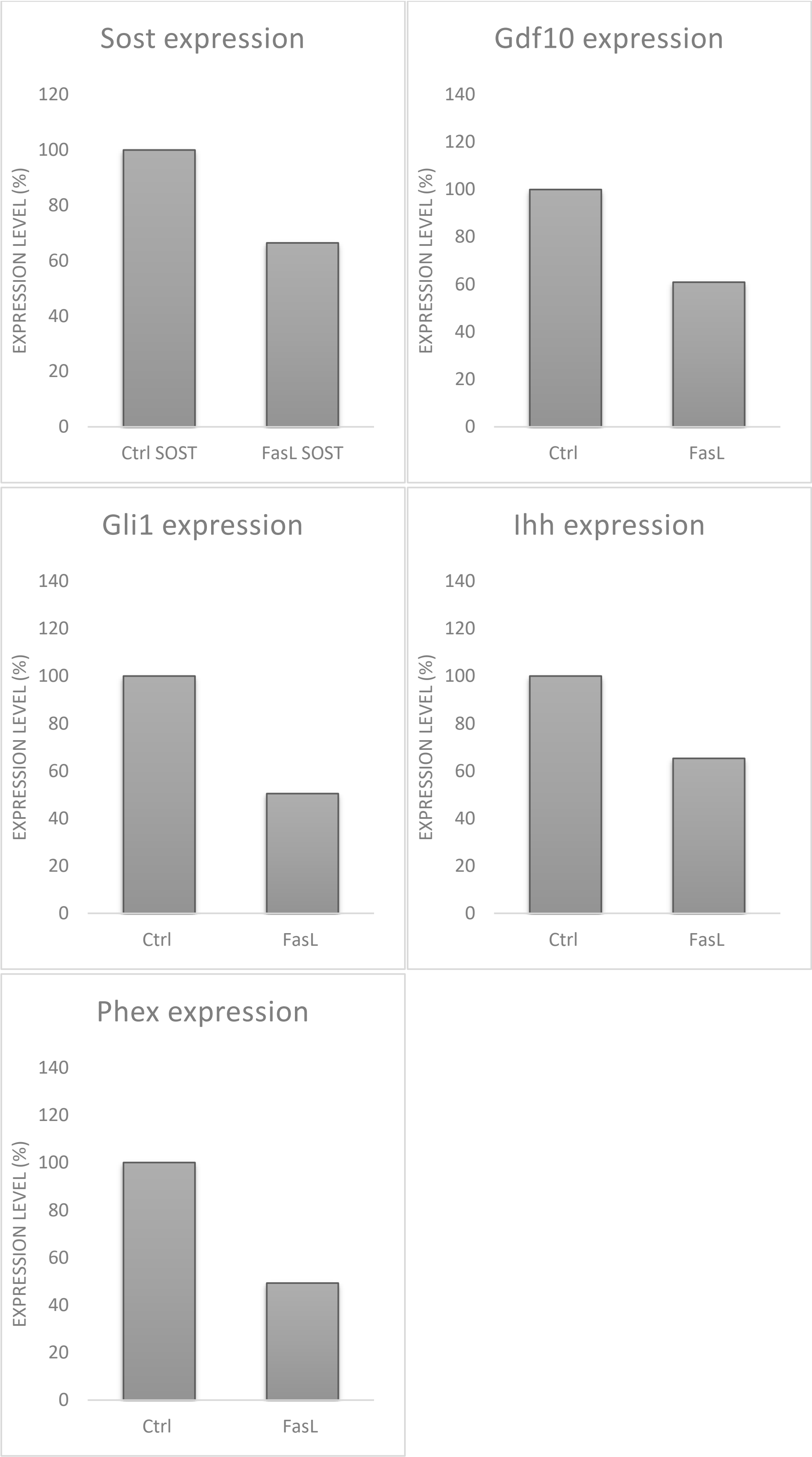

Figure S2: Expression of Sost (A), Gdf10 (B), Gli1 (C), Ihh (D), and Phex (E) in calvarial cells after FasL stimulation.

A	Ctrl	FasL	FasL+OPh
percentage of non-viable cells	1 %	18 %	6 %
average	0.01	0.18	0.06
sd	0.02	0.03	0.07
t-test		0.000075	0.021<br>0.225

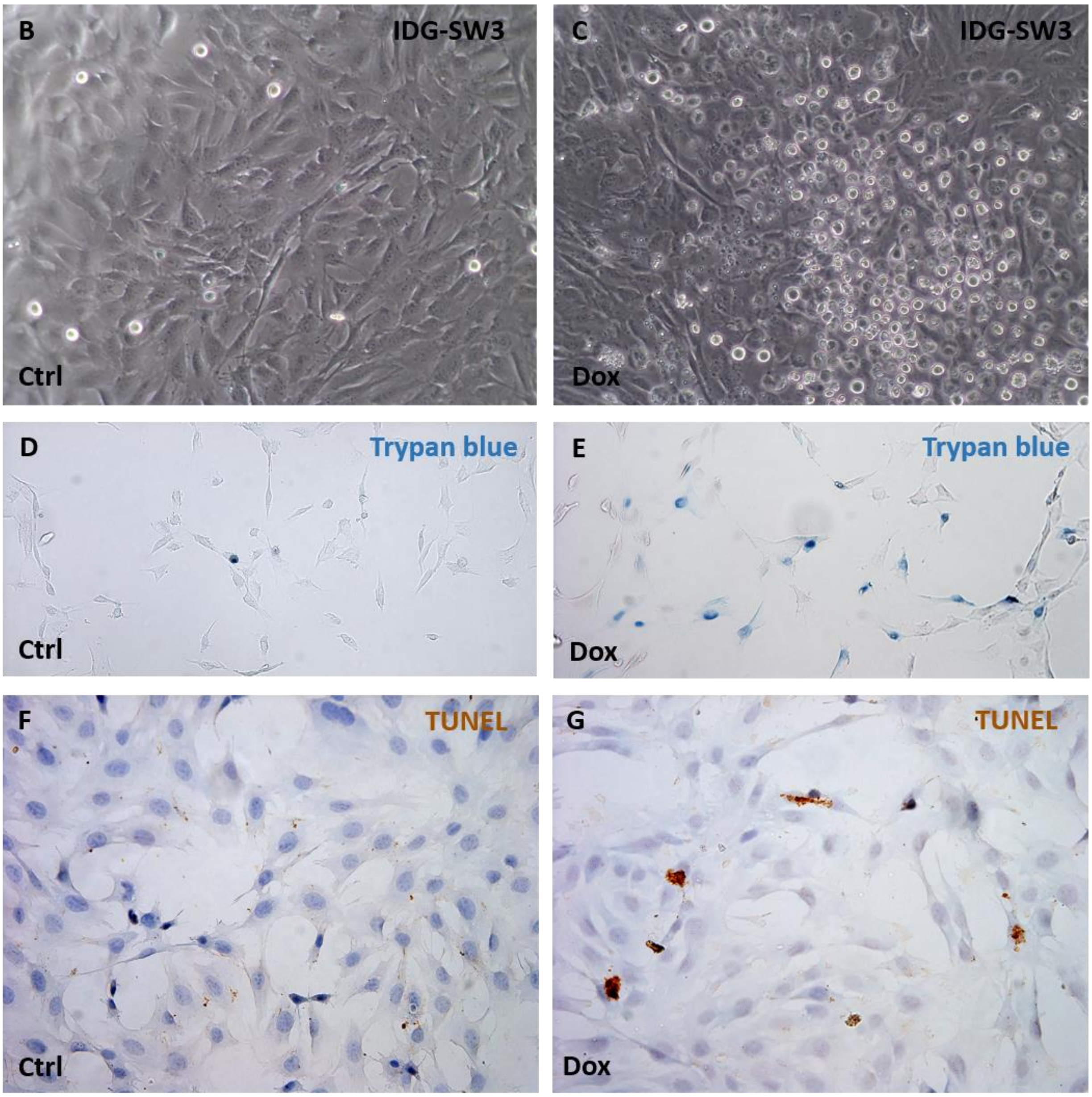

Figure S3: Quantification of non-viable IDG-SW3 cells in control, FasL and FasL+OPh treated cultures counted as a percentage of trypan blue-positive cells, cells were counted in four independent fields of vision (A). Positive control of apoptosis was performed by treatment of 5 µM doxorubicin (B, C). After 6 hours, treated cells (C) began to die, left the surface, and floated in the medium. The remaining doxorubicin treated cells showed decreased viability (E) compared to untreated cells (D). To confirm ongoing apoptosis, TUNEL assay, a method for detection of more pronounced stages of apoptosis, was used (F, G). Ctrl: control, Dox: doxorubicin, OPh: Q-VD-OPh inhibitor.
